# Molecular Surveillance Reveals *F*-Gene Mutations and Constrained *G*-Gene Evolution in Human Respiratory Syncytial Virus: Implications for Vaccine Efficacy in Saudi Arabia

**DOI:** 10.3390/vaccines13121245

**Published:** 2025-12-15

**Authors:** Mohamed A. Farrag, Ibrahim M. Aziz, Abdulaziz M. Almuqrin, Noorah A. Alkubaisi, Reem M. Aljowaie, Asma N. Alsaleh, Fatimah N. Alanazi, Adel A. Abdulmanea, Fahad N. Almajhdi

**Affiliations:** 1 Department of Botany and Microbiology, College of Science, King Saud University, Riyadh 11451, Saudi Arabia; iaziz@ksu.edu.sa (I.M.A.); nalkubaisi@ksu.edu.sa (N.A.A.); raljowaie@ksu.edu.sa (R.M.A.); asmalsaleh@ksu.edu.sa (A.N.A.); 445205516@student.ksu.edu.sa (F.N.A.); aabdulmanea@ksu.edu.sa (A.A.A.); 2 Department of Clinical Laboratory Sciences, College of Applied Medical Sciences, King Saud University, Riyadh 12372, Saudi Arabia; aalmuqrin@ksu.edu.sa

**Keywords:** human respiratory syncytial virus, hRSV vaccine, vaccine effectiveness, sequence/phylogenetic analysis, Saudi Arabia, ON1

## Abstract

Background/Objectives: Human Respiratory Syncytial Virus (HRSV) is a major global cause of acute lower respiratory infections in children. With recent approval of pre-fusion F protein-based vaccines and monoclonal antibodies, ongoing molecular surveillance is critical. This study examined HRSV molecular epidemiology and evolution in Riyadh, focusing on mutations in the attachment (G) and fusion (F) glycoproteins and their potential impact on vaccine efficacy. Methods: Nasopharyngeal aspirates (NPAs) (200 samples) were collected from pediatric patients. HRSV-positive samples were typed, and the *G* gene hypervariable region and *F* gene were sequenced. Sequence and phylogenetic analyses were performed to identify circulating genotypes and amino acid substitutions. Results: HRSV was detected in 15% of samples, with HRSV-B slightly predominant over HRSV-A. Infants aged 2–5 months had the highest incidence rate of infection. The ON1 subgenotype remained dominant. The duplicated region of the *G* gene showed constrained evolution, with 18 variable and 6 conserved residues over 13 years. In the F protein, HRSV-A isolates exhibited high conservation, with only three amino acid substitutions in antigenic sites (Ø and II). Sites III, IV, and V remained fully conserved. In contrast, HRSV-B isolates displayed eight substitutions in antigenic sites, including six in site II (palivizumab-binding epitope). Conclusions: Given the highly effective HRSV prophylactics, including the approved vaccines and monoclonal antibodies, these mutations raise critical concerns regarding vaccine efficacy against HRSV-B. These findings underscore the necessity of sustained, seasonal molecular surveillance to monitor the emergence of variants and provide a molecular basis for further clinical studies.

## 1. Introduction

Human Respiratory Syncytial Virus (HRSV), also referred to as Human Orthopneumovirus (HOPV), is one of the leading causes of acute lower respiratory tract infections (RTIs) in infants, children, immunocompromised individuals, and elderly individuals [1,2]. Because long-lasting immunity cannot be established, recurrent HRSV infections are common, which impedes the development of viable vaccine candidates [3]. HRSV infections are associated with a significant rate of morbidity and mortality across the globe. According to recent estimates, HRSV infections are associated with 33 million confirmed cases of acute RTI worldwide. In addition, it was reported that HRSV infection is associated with nearly 3.6 million hospitalizations and 26,300 hospital deaths. It is estimated that HRSV infection causes more than 100,000 deaths in children younger than five years [4].

HRSV is classified within the *Pneumovirus* genus of the *Pneumoviridae* family. It is a single-stranded negative-sense RNA virus with a genomic size ranging from 15,191 to 15,226 nts long that encodes eleven structural and nonstructural proteins, including the major glycoproteins on its surface and the fusion envelope (F) and attachment (G) proteins [5]. The G and F proteins play critical roles in viral infection, as G facilitates virion attachment to the host cell membrane, whereas F mediates the fusion step [6]. HRSV is classified into two main groups, A and B, based on genetic variations in the G glycoprotein. HRSV-A currently comprises at least 14 genotypes (GA1–GA7, SAA1, NA1–NA4, ON1, CB-A, etc.), whereas HRSV-B includes more than 30 genotypes [7,8].

The F protein is primarily synthesized as the F0 precursor (574 amino acids). It undergoes cleavage by a furin-like host protease to yield two subunits (F1 and F2) and pep27 polypeptides [9]. The resulting mature F protein is a homotrimer, with F1 being essential for membrane fusion [10]. Structurally, the protein is known to transition between a metastable, ‘lollipop’-shaped prefusion conformation found on the virion membrane and a stable, cone-shaped postfusion form characterized by a 6-helix bundle [11]. Due to its high sequence conservation, the F protein is a critical target for both cytotoxic T lymphocytes [12] and for neutralizing antibody and vaccine development. Six antigenic sites named Ø, I, II, III, IV, and V have been mapped within the F protein [13]. The antigenic site Ø is exclusively found in the prefusion state [14]. The antigenic site II, located between amino acids 262 and 275, serves as the binding domain for the therapeutic neutralizing antibodies such as palivizumab and motavizumab, where mutations can lead to drug resistance [15].

HRSV is characterized by a high mutation rate, leading to the continuous emergence of new genotypes with distinct clinical manifestations in infected individuals [16]. The most recent example of this is the HRSV-A genotype ON1, with a duplication of 72 nucleotides (nt) in the second hypervariable region of the *G* gene, which first appeared in Ontario, Canada, in 2010. Since then, multiple studies have reported the emergence of this genotype in many countries [17,18,19]. In Saudi Arabia, our group previously reported that most HRSV infections during the winter seasons of 2007/08 and 2008/09 in Saudi Arabia belonged to the HRSV-A group, and we observed a genotype shift from NA1 to ON1 [20].

The history of HRSV prevention is characterized by a significant early setback with the formalin-inactivated respiratory syncytial virus (FI-RSV) vaccine in the 1960s, which led to vaccine-enhanced respiratory disease and suspended vaccine development for decades [3]. For over twenty years, the primary prophylactic tool was the monoclonal antibody palivizumab (Synagis), which provides passive immunity by targeting conserved antigenic site II on the F protein in high-risk infants [21]. However, the landscape of HRSV prevention has been fundamentally changed by the recent approval of stabilized prefusion F protein-based vaccines, Arexvy (GSK) and Abrysvo (Pfizer) [22,23]. These vaccines, approved in 2023, represent a major scientific breakthrough, with Arexvy being the first to gain approval for older adults and Abrysvo being approved both for older adults and for maternal immunization to protect infants [24]. The efficacy of these F protein-targeting prophylactics, including the recent long-acting monoclonal antibody, nirsevimab, is directly dependent on the antigenic stability of the circulating strains. Although the F protein is considerably conserved among different HRSV types, a single amino acid change, particularly within the antigenic sites, can interfere with the efficacy of monoclonal antibodies and vaccine effectiveness [25]. Continuous molecular surveillance is therefore essential to detect emerging variants that could compromise the effectiveness of these newly introduced prophylactics.

Despite the worldwide rollout of HRSV F-based vaccines and monoclonal antibody therapeutics, comprehensive data on the long-term molecular epidemiology of HRSV-A in Saudi Arabia remain inadequate. The evolutionary kinetics behind the sustained dominance of the ON1 genotype and the antigenic evolution of the F protein have not been systematically investigated in Saudi Arabia. Therefore, the current study aimed to (i) describe the molecular epidemiology and circulation pattern of HRSV-A in Riyadh, Saudi Arabia, over multiple seasons, (ii) elucidate the genetic factors contributing to the continued predominance of the ON1 genotype, and (iii) examine the antigenic stability of the F protein in contemporary Saudi HRSV isolates in the context of the recently approved vaccines and monoclonal antibody therapeutics. To achieve these objectives, NPAs were collected from hospitalized children and screened for HRSV. The *G* and *F* genes of HRSV-A strains were amplified and sequenced for sequence and phylogenetic analysis.

## 2. Materials and Methods

### 2.1. Acquisition of Clinical Samples and Ethics Statement

After the parents of the children provided a written informed consent form and the Research Ethics Committee (RES) at King Saud University, Riyadh, Saudi Arabia, approved the study (Ethics Reference No. E-141326/IRB), 200 NPA samples of children at King Khalid University Hospital who had symptoms of respiratory tract infection were collected during the winter of 2022–2023. The Virology Research Laboratory (VRG) at King Saud University’s College of Science collected the samples, combined them with two milliliters of the viral transport medium, and sent them right away in cooling boxes. After that, the samples were divided into smaller portions and stored at −80 °C until analysis.

### 2.2. HRSV Detection, Typing, and Amplification of G and F Gene Sequencing Fragments

A QIAamp Viral RNA Extraction Kit (Qiagen, Hilden, Germany) was used to extract viral RNA from clinical samples. A One-Step RT-PCR Kit (Qiagen, Hilden, Germany) and the following primer set were used: HRSV-U-F (5′-GGA ACA AGT TGT TGA GGT TTA TGA ATA TGC-3′) and HRSV-U-R (5′-CTT CTG CTG TCA AGT CTA GTA CAC TGT AGT-3′). PCR typing was attempted via the same PCR kit and the following primer sets: HRSV-A [HRSVA-F (5′-GAT GTT ACG GTG GGG AGT CT-3′) and HRSV-A-R (5′-GTACACTGTAGTTAATCACA-3′)] and HRSV-B [HRSV B-F (5′-AATGCTAAGATGGGGAGTT-3′) and HRSV B-R (5′-GAAATTG AGTTAATGACAG-3′)] [26]. The PCR program included reverse transcription for 30 min at 50 °C, initial denaturation at 95 °C for 15 min, 35 cycles of denaturation at 94 °C for 30 s, annealing at 52 °C for 30 s, and extension at 72 °C for 2 min, followed by a final extension step at 72 °C for 10 min. The reaction was carried out in a GeneAmp 9700 thermal cycler (Thermo Fisher Scientific, Waltham, MA, USA). The amplified products were compared to a 100 bp DNA ladder (Qiagen, Hilden, Germany) and visualized on an agarose gel stained with 1% ethidium bromide.

The G ectodomain encompassing the two HVRs and the full-length *F* gene was amplified via the One-Step Ahead RT-PCR Kit (Qiagen, Hilden, Germany), which was carried out in a GeneAmp 9700 thermal cycler. The following two primer sets were used for the *G* gene [HRSVA-G-F1 (5′-CTC GAG TCA ACA CAT AGC ATTC-3′), HRSVA-G-F1 (5′-GTT GGA TTG TTG CTG CAT ATG-3′), HRSVA-G-F2 (5′-CAAGATGCAACAAGCCAGATC-3′), and HRSV-G-R2 (5′-ACTG CAC TGC ATG TTG ATT G-3′)]. Three primer sets were used to amplify the whole *F* gene [HRSVA-F-F1 (5′-GGC AAA TAA CAA TGG AGT TG-3′); HRSV-F-R1 (5′-CTC CAT TTG ATA AGC TGA CTAC-3′); HRSVA-F-F2 (5′-GTT AGG TGT TGG ATC TGC AATC-3′); HRSV-F-R2 (5′-CAT AGC ATG ACA CAA TGG CTC-3′); and HRSVA-F-F3 (5′-GAG GAT GGT ACT GTG ACA ATG-3′) and HRSV-F-R3 (5′-GCA AGG ATT CCT TCG TGAC-3′)]. DNA bands corresponding to the expected size were retrieved from the gel via the Illustra™ GFX PCR DNA and Gel Band Purification Kit (GE Healthcare, Chicago, IL, USA). The amplified fragments were sequenced on both strands via Macrogen, Inc. (Seoul, Republic of Korea). Raw sequence data were edited via the BioEdit program, version 7.0 (Ibis Biosciences, Carlsbad, CA, USA), and assembled via the Edit sequence tool of the MegAlign program, Lasergene software, version 3.18 (DNAStar, Madison, WI, USA). Only 9 sequences were selected for sequence and phylogenetic analysis on the basis of their sequence heterogeneity. The final assembled sequences were added to the GenBank database with accession numbers [G: PP454562–PP454570) and (F: PP454571–PP454579)].

### 2.3. Nucleotide and Deduced Amino Acid Sequence Analysis of the G and F Genes/Proteins

To understand the evolution and diversity of HRSV-A in Riyadh over time, the 9 sequences of the *G* gene from the winter season of 2022/23 were aligned with Riyadh strains from 6 previous winter seasons: 2007/08 (*n* = 4), 2008/09 (*n* = 5), 2014/15 (*n =* 6), 2015/16 (*n* = 5), 2019/20 (*n* = 4) and 2021/22 (*n* = 3). A total of 61 international strains used for phylogenetic analysis were retrieved from GenBank databases based on the following criteria: (i) a broad range of geographical regions and collection years; (ii) representative strains from all genotypes to ensure a comprehensive evolutionary context; (iii) representative of the ON1 sequences that described in the earliest reports; and (iv) prototypes as reference strains (AF065257, M17212, KT992094, AF233917, and KU316106). Both the nucleotide and deduced amino acid sequences were aligned via ClustalW, the MegAlign program of Lasergene software, version 3.18 (DNAStar, Madison, WI, USA). Changes in protein length, sequence gaps, insertions and deletions, and synonymous and nonsynonymous mutations were considered in the analysis. In addition, genotype-specific amino acids were determined. Similarly, the nucleotide and deduced amino acid sequences of the full-length *F* gene (seasons: 2008/09 and 2022/23) were aligned against 43 international strains retrieved from Gene bank database. These strains were selected to cover a broad range of temporal and geographical regions. Crucially, the *F* gene of the HRSV-B type was included in the study to assess the potential vaccine efficacy against this subtype. Furthermore, 4 reference strains (AF065257, M17212, KT992094, and AF233917), which are the most used in vaccine design, were incorporated into the comparison. The *F* gene of strain A2, widely used for vaccine design, was set as the consensus sequence. Mutations at both the nucleotide and amino acid levels were determined, and amino acid changes within the five antigenic sites of the F protein were recorded.

### 2.4. Construction of Phylogenetic Trees and Genotype Distribution

The strains detailed in the previous section were utilized to generate two phylogenetic trees. The first tree included a total of 101 sequences (61 international + 40 local) and was constructed based on the 2nd HVR of the *G* gene. The second tree included 55 strains (43 international + 12 local) and was constructed based on the full-length *F* gene. Both trees were inferred using the Maximum Likelihood method via MEGA 6.0 (Pennsylvania State University, University Park, PA, USA). The accuracy of the tree topology was evaluated by performing 1000 bootstrap replicates.

### 2.5. Glycosylation Profiles of the G and F Proteins

Deduced amino acid sequences (Riyadh and international strains) of both the G and F proteins were used to determine potential O- and N-glycosylation sites via Net-N-glyc 1.0 and Net-O-glyc 3.1, respectively [27,28]. Conserved and new potential sites for N- and O-glycosylation were identified.

## 3. Results

### 3.1. Prevalence of HRSV in Study Subjects

Among the 200 NPAs tested for HRSV, 30 (15%) were positive; 14 (7%) were HRSV-A, and 16 (8%) were HRSV-B (Table 1). The screening results indicated a slight predominance of HRSV-B over HRSV-A. Age-group analysis revealed that the prevalence of HRSV-A was considerably greater (6.74%) (*p* < 0.05) in the 2–5-month age group. The sex-based analysis revealed that male-infected children had a greater incidence of HRSV-A infections (7.69%) than female-infected children (6%).

### 3.2. Sequence Analysis of the G Gene/Protein

The kinetics of HRSV-A evolution were analyzed over 7 winter seasons: 2007/08, 2008/09, 2015/16, 2016/17, 2019/20, 2021/22, and 2022/23. The analysis was based mainly on the two HVRs of the attachment *G* gene, which ranged in length from 615 nt (genotypes GA-1 to GA7 and SAA1) to 687 nt (subgenotype ON1). Nucleotide sequence analysis was performed by aligning the two HVRs of the Saudi strains from the winter season (2022/23) and the 6 previous winter seasons with their international counterparts available in the GenBank database. An overview of the nucleotide sequence alignment report revealed no sequence abnormalities or sequence gaps. The subgenotype ON1 possesses a unique sequence feature that distinguishes it from the other genotypes, which is the insertion of a duplicate region of 72 nucleotides at the C-terminus of the second HVR. This insertion resulted in an increase in the length of the *G* gene from 894 to 966 nucleotides. This feature is shared by all ON1 strains circulating worldwide. No year-specific mutations among the different HRSV-A genotypes were recorded. The overall sequence heterogeneity between Saudi ON1 strains (winter season: 2022/23) ranged from 0.4 to 8.6% and from 2.7% to 10.6% at the nucleotide and amino acid levels, respectively. However, the degree of sequence heterology between the Riyadh ON1 and GA2 strains ranged from 7.4% to 13.9% and from 5.1% to 16.3% at the nucleotide and amino acid levels, respectively.

**Figure 1:**
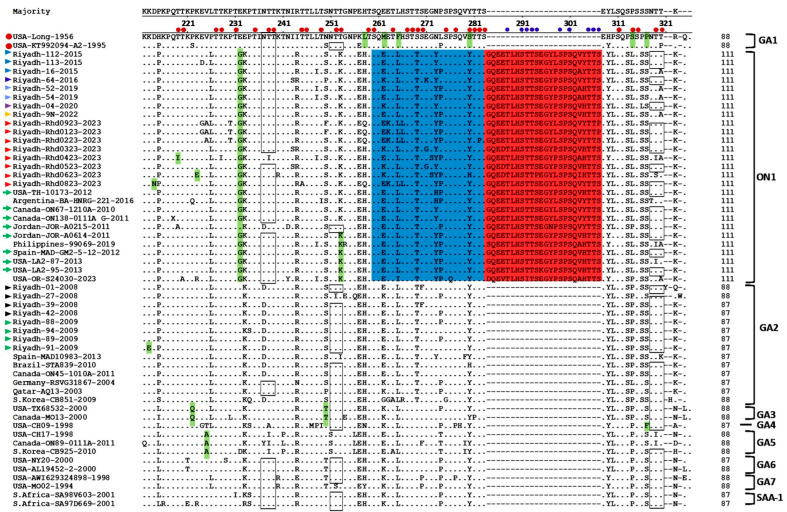
Deduced amino acid alignments of the 2nd HVR of HRSV-A G protein. The figure presents different virus genotypes, and the alignment was performed via the Clustal W method within the MegAlign program (DNAstar). The sequence alignments were performed in comparison with the reference sequences of the prototype strains (USA-Long-1956 and strain A2) indicated by red circles at the left of the figure. Dots represent identical amino acid residues. Genotype-specific amino acid residues are highlighted in blue, and the duplicated region is highlighted in green. Potential sites of N-glycosylation are shown within rectangles, and O-glycosylation sites are indicated by small red circles and blue circles refer to new glycosylation sites in the duplicated region. The colored triangles at the left of the figure refer to the Riyadh strains from different winter seasons. Green arrows refer to the representative ON1 sequences that were described in the earliest reports.

The Saudi ON1 strains of the winter season (2022/23) presented a total of 130-point mutations, which represented 19% of the sequenced fragment. Among these mutations, 66 mutations were nonsynonymous and resulted in changes in their corresponding amino acid residues. Intensive amino acid analysis of the 2nd HVR revealed several genetic characteristics of the HRSV genotypes. In addition to the duplicate region (Figure 1), a total of 16 (18.6%) amino acid residues were conserved among the different genotypes. Some amino acids were specific to individual genotypes, including 233E, 258 L, 262 M, 265F, 280S, 290S and 293P for genotype GA1; 222Q and 250T for genotype GA3; and 225A for genotype GA5. All ON1 strains, including the Riyadh strains, presented an insertion of 24 amino acids between residues 283 and 284, of which 23 amino acids were duplicated, as highlighted in Figure 1. In addition, ON1 strains have two specific amino acids: 232G and 253K. The GA2 genotype strains, including ON1, share one common specific amino acid at position 269T (Figure 1). The Saudi ON1 strains presented the following specific amino acids: 230T for Riyadh-01-2023, 243S for Riyadh-03-2023, 219I and 227I for Riyadh-04-2023, and 213 N for Riyadh-08-2023.

### 3.3. Evidence of Constrained Hyper-Evolution in ON1 G Gene Duplication

The duplicated region (nts: 811–882) of Saudi ON1 strains (5 winter seasons) has been aligned against 100 international ON1 strains since 2011. The ON1 prototype, ON138-0111A (accession number: JN257694), was set as the consensus sequence. The duplicate regions of the 9 Riyadh ON1 strains (winter season 2022/23) presented 18 amino acid substitutions. However, the Riyadh ON1 strains (winter season 2019/20, 2020/21) presented 17 amino acid changes and 14 amino acid changes in the Riyadh ON1 strains in the winter seasons of 2014/15 and 2015/16. The duplicated region of all ON1 strains had 6 amino acids (285Q, 288T, 292T, 295E, 302Q, and 306T) that remained unchanged across 13 years. This constrained evolution suggests a strong functional requirement for the conserved residues, while the variable sites are under intense immune pressure (Figure S1).

### 3.4. Sequence Analysis of the F Gene/Protein

**Figure 2:**
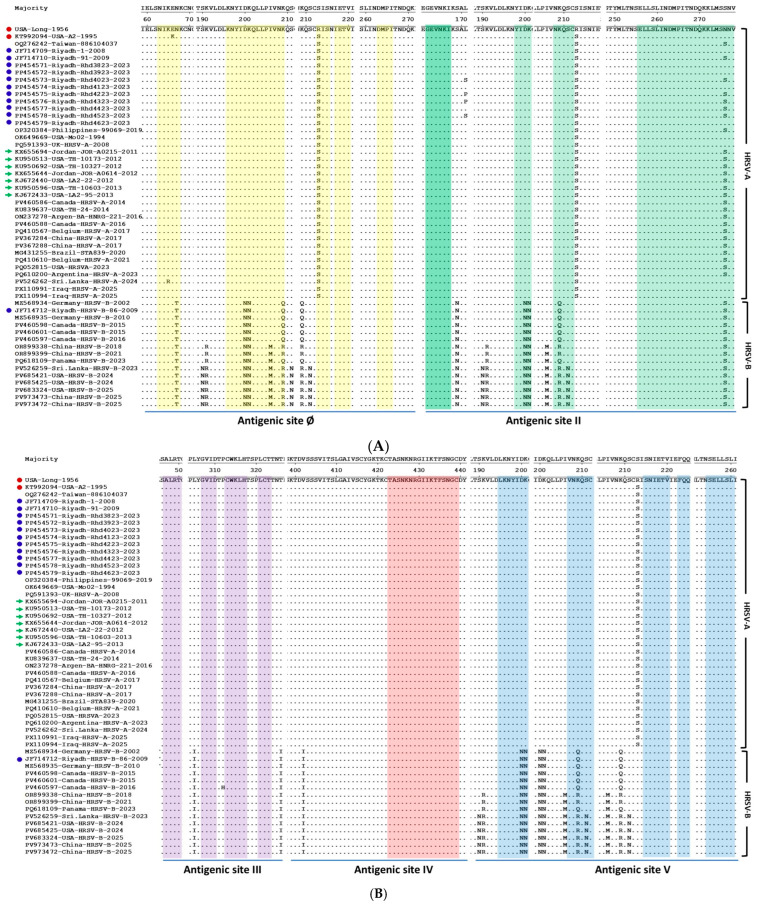
Multiple sequence alignment of the deduced amino acid sequences of the HRSV F protein from Riyadh isolates and global strains. The alignment compares the F protein sequences of HRSV-A and HRSV-B isolates, including those from Riyadh, against those of the reference strains (USA-Long-1956 and Strain A2). Dots indicate identity with the reference sequence, whereas letters denote amino acid substitutions. The five major antigenic sites (Ø, II, III, IV, and V) are highlighted and labeled at the bottom. The Riyadh HRSV-A strains are denoted by blue circles, reference strains in red circles and green arrows refer to the representative ON1 sequences that were described in the earliest reports. (**A**) amino acid residues mapped within antigenic sites Ø and II. (**B**) amino acid residues mapped within antigenic sites III, IV, and V. Highlighted colored rectangles indicate amino acid residues for each site.

The nucleotide and deduced amino acid sequences of the HRSV-A and HRSV-B F protein were aligned against the reference strains (USA-Long-1956, Strain A2), which are the most commonly used for vaccine design. This comparative analysis was specifically conducted to identify mutations within the five major antigenic sites to assess the potential impact of these amino acid changes on viral antigenicity and the effectiveness of current or future vaccine candidates. At the nucleotide sequence level, in comparison with the consensus strain, 112 nucleotide mutations were reported in HRSV-A strains, and 309 were reported in HRSV-B strains. Analysis of the deduced amino acid sequences of the HRSV-A and HRSV-B F protein (Figure 2A,B) from Riyadh isolates revealed distinct patterns of variation across the five major antigenic sites in the prefusion F protein (Ø, II, III, IV, V). The HRSV-A isolates displayed a total of 24 amino acid changes, with only three substitutions mapping to the defined antigenic sites: two at Site Ø (K65R and R213S) and one at Site II (N276S). Notably, the three antigenic sites III, IV, and V showed no amino acid changes in the HRSV-A strains, indicating a high degree of conservation in these regions, a finding consistent with the visual stability observed in the global HRSV-A lineage.

In contrast to HRSV-A, the HRSV-B subgroup, which visually exhibits greater overall sequence diversity, presented a significantly greater number of total amino acid changes (*n* = 63) in the Riyadh isolates. Eight of these changes are located within the antigenic sites, with six changes specifically reported in antigenic site II (the palivizumab-binding epitope): D200N, K201N, K209Q, K209R, S211N, and N276S. The HRSV-B isolates also presented a significantly greater number of total amino acid changes (*n* = 63). Eight of these changes are located within the antigenic sites (N67T, D200N, K201N, I206M, K209Q, K209R, S211N, and N276S). Due to the overlapping of these antigenic sites, reported amino acid changes may affect more than one site. The remaining two changes are N67T (Site Ø) and I206M (Site II). Due to the overlapping nature of these antigenic sites, some reported amino acid changes may affect more than one site. Notably, both HRSV-A and HRSV-B isolates were completely conserved across antigenic sites III and IV, suggesting these regions are under strong functional or structural constraint. The observed accumulation of mutations in the HRSV-B antigenic sites, particularly Site II, highlights a significant difference in the evolutionary dynamics between the two subgroups and suggests a potential mechanism for immune evasion, a conclusion supported by the visual evidence of increased substitution frequency in the global HRSV-B strains (Figure 2A,B).

Amino acid substitutions in both HRSV-A and HRSV-B strains are concentrated near key antigenic sites, suggesting potential impacts on immune recognition. In HRSV-A strains, specific changes were reported near antigenic site II (L171S/P and R213S) and site IV (R213S). The HRSV-B strains displayed a broader mutational profile, with several substitutions clustered across multiple sites: S190N, K191R, and S211N were observed near site Ø; S169N and I206M were found near site II; L305I, P311H, and T326I were observed near site III; and S190N, K191R, and I206M were identified near site V.

### 3.5. Phylogenetic Analysis

**Figure 3:**
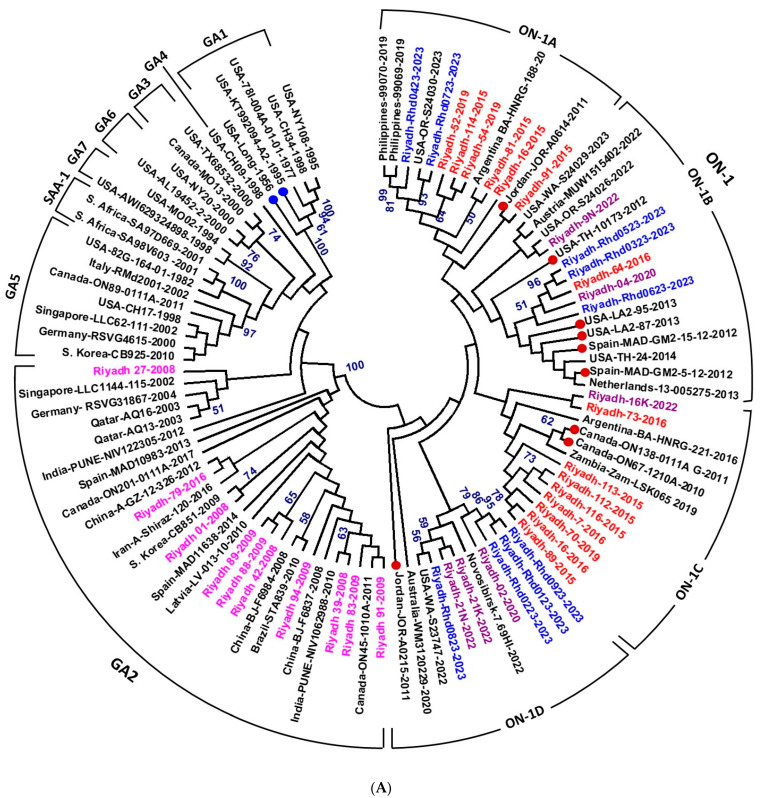

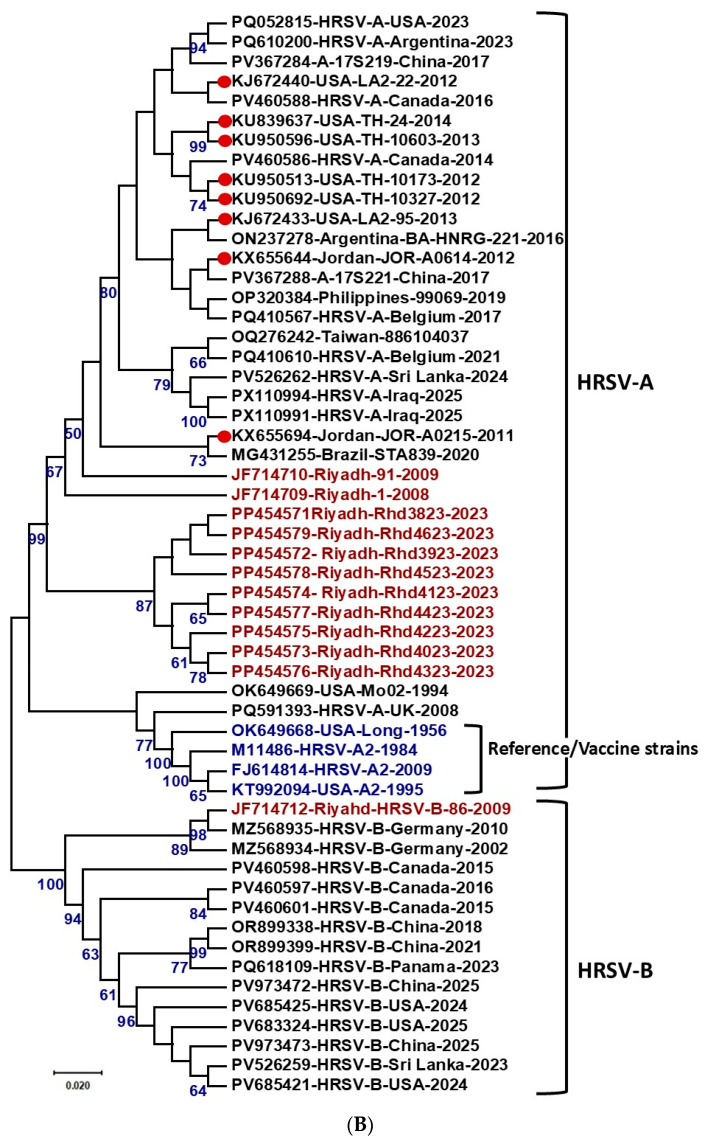
Phylogenetic analysis of HRSV strains from Riyadh, Saudi Arabia. (**A**) Phylogenetic analysis of HRSV-A strains based on the second hypervariable region (2nd HVR) of the *G* gene. A total of 101 sequences representing different HRSV-A genotypes, including Riyadh strains, were aligned using ClustalW, and a phylogram was generated using the maximum likelihood method in MEGA 6.06. Bootstrap values >50% are shown. HRSV-A genotypes and subgenotypes are indicated at the periphery. Riyadh strains from seven winter seasons are color-coded and distributed in the NA2 subgenotype (seasons 2001/08 and 2008/09), while strains from five winter seasons (2014–2023) clustered within ON1 subgenotype lineages. (**B**) Maximum likelihood phylogenetic tree based on full-length *F* gene sequences of HRSV-A and HRSV-B strains. The tree is rooted with reference/vaccine strains A2 (M11486, FJ148414 and KT992094). Major clades for HRSV-A and HRSV-B subgroups are delineated, with bootstrap support values >60% indicated at key nodes. Riyadh isolates are highlighted in red font, and the reference/vaccine strains in blue font.

Phylogenetic analysis of the 2nd HVR *G* gene resulted in the clustering of the HRSV-A strains into 8 genotypes: GA1–GA7 and SAA1. The GA1 clade includes the extinct strains identified early in the USA. The GA2 clade is divided into two subclades: the ON1 subclade with four cluster groups (ON1A to ON1D) and the other GA2 strains subclades (Figure 3A). The Saudi strains identified in the 2 winter seasons of 2007/08 and 2008/09 were clustered into the GA2 genotype, whereas strains of the 5 winter seasons of 2014/15, 2016/17, 2019/20, 2021/22, and 2022/23 were clustered within the ON1 genotype with no special preference for distinct subgenotypes (Figure 3A). The strains are scattered and clustered within genotypes on the basis of their genetic relatedness, with no effect on the period.

The phylogenetic analysis of the full-length *F* gene (Figure 3B) clearly demonstrated the evolutionary relationships of the isolates. The significant sequence heterogeneity between the two major subgroups results in the robust separation of all strains into two distinct clades: HRSV-A and HRSV-B. The historical reference/vaccine strains, OK649668, M11486 and FJ614814 (strain A2), are positioned at the base of the HRSV-A clade, confirming their ancestral status within this subgroup. The nine HRSV-A isolates from Riyadh form a distinct, highly supported monophyletic cluster (99% bootstrap value) that is nested within the larger HRSV-A clade, demonstrating a close evolutionary relationship with the reference strains and other contemporary global HRSV-A isolates. This clustering indicates that while the Riyadh HRSV-A strains constitute a locally evolving lineage, they share a common ancestor with the M11486 vaccine strain. In contrast, the single HRSV-B isolate from Riyadh is located in a separate HRSV-B clade, confirming the genetic distance between the two subgroups.

### 3.6. Prediction of N- and O-Glycosylation Sites of HRSV Genotypes

The deduced amino acids (229 aa for ON1 strains and 205 aa for the other genotypes) were analyzed for possible sites for O- and N-glycosylation. For the GA1 strains, 5 sites (103, 135, 237, 251, and 294) were predicted as potential sites for N-linked glycosylation. The GA2 genotype has the same N-linked glycosylation sites as GA-1 does. However, some GA2 strains, such as Brazil-STA839-2010, lack site 237 because of the N237D mutation. Some Saudi GA2 strains from the winter seasons of 2007/08 and 2008/09, such as Riyadh-27-2008, presented six potential N-glycosylation sites. The 9 Saudi ON1 strains from the winter season (2022/23) presented variable numbers of N-linked glycosylation sites, ranging from 2 in Riyadh-06-2023 and -07-2023 to 4 in Riyadh-08-2023. In addition, these strains have two conserved N-glycosylation sites at 103 NLSG and 237 NTTK compared with three conserved N-glycosylation sites (103 NLSG, 135 NTT, and 237 NTTK) in the winter seasons of 2014/15 and 2016/17.

Saudi ON1 strains harvested in the winter of 2021–2022 presented 3–5 sites for N-glycosylation. However, ON1 strains from the winter seasons of 2007/08 and 2008/09 presented 3 conserved N-glycosylation sites at residues 103, 135, and 237. The number of these sites in international ON1 strains ranged from 3 sites in the Philippines-99071-2019 to 5 sites in Novosibirsk-7.69Hl-2022. Site 251, the most common N-glycosylation site in HRSV genotypes, is lacking in ON1 strains because of the mutation T253K, a characteristic mutation of this subgenotype (Figure 1). For the GA5 and GA7 genotypes, 5 potential N-glycosylation sites were recorded (103, 135, 237, 250 (GA5)/251 (GA7), 294).

The G protein is rich in serine and threonine residues, which range from ~33S/58T in GA2-7 to 37S/64T in the ON1 subgenotype and represent 30.5% and 31% of the whole G protein length, respectively. In the analyzed fragment, the number of potential O-linked glycosylation sites generally ranged from 56 in Panama-PAN22A-2011 to 77 in Austria-MUW1515402-2022. The number slightly increased to 78 sites in Riyadh-05-2023. In general, the number of O-linked glycosylation sites increased in ON1 strains (82 sites in some strains; G score 0.5–0.99) due to the insertion of 24 aa, the duplicate region (Figure 1). In contrast to the G protein, the F protein of all strains, including Saudi strains, presented 5 conserved sites (27, 70, 116, 126, and 500) for N-linked glycosylation (Figure S2), with few exceptions, with only 4 sites, such as UK-440104002-2021 and Riyadh-RSVAF44-2023. In addition, no O-linked glycosylation sites were predicted in the F protein in most of the strains, including Saudi strains. Only a few strains displayed one or more potential sites for O-glycosylation: USA-RSV-A-human-OR-S24493-2023 (1 site), Riyadh-91-2009 (3 sites), Spain-SE01A-0064-V02-2018 (3 sites), USA-strain long-1956 (3 sites), and USA-A2-1995 (3 sites).

## 4. Discussion

HRSV has long been recognized as a common cause of lower respiratory tract infections in humans of different age groups. Disease severity and mortality are common in neonates, elderly individuals, and immunocompromised patients. Since its discovery more than 6 decades ago, no fully protective vaccine has been developed. In the present study, HRSV was detected in 30 (15%) of the 200 NPA samples collected during the two winter seasons of 2021–22 and 2022–23. In terms of age group, the incidence rate was high (25%) in infants (<2 months), followed by 15.38% (≥12 months), 13.33% (6–11 months), and 11.24% in infants aged 2–5 months. These findings are consistent with our previous studies in Riyadh [20,29,30], reports from other parts of Saudi Arabia [31,32,33,34], nearby countries [35,36,37,38], and worldwide records [39,40,41,42]. The high incidence rates in neonates and younger infants could be attributed to the immature immune response and waning levels of maternal antibodies [43,44]. In the study group, HRSV-B slightly predominated over HRSV-A. This finding was reported in several studies where both types alternately predominated during epidemics [38,45,46].

Recurrent infections, even with the same genotype, are common [8,47]. This could be because the virus continually produces new genotypes and variants to evade preexisting immunity [16,48]. Therefore, monitoring virus evolution and linking disease severity to circulating genotypes are priorities for alleviating disease symptoms and reducing mortality rates. During epidemics, several genotypes of HRSV-A circulate concurrently, and dynamic replacement of the existing genotypes has been reported between epidemics on a spatial and temporal basis.

The two HVRs of the *G* gene were used for nucleotide and deduced amino acid analysis. However, the 2nd HVR region was used for phylogenetic analysis, which is reliable and has been reported in several studies for both types (A and B) of HRSV worldwide [49,50,51]. The evolutionary dynamics of HRSV witness the extinction, replacement and co-circulation of different genotypes. For example, the GA1 genotype became extinct approximately 44 years ago [52]. The predominant genotypes until 2007 were GA2 and GA5 [53,54], and within 7 years, GA2 replaced GA5 [55]. The GA2 genotype continues to generate new subgenotypes (e.g., NA-1, NA-2, and CB-A), which spread to all countries worldwide [20]. Among these subgenotypes, NA-1 and NA-2 were replaced within 2 to 4 years by the subgenotype ON1. ON1 was first reported in Ontario, Canada [18], and subsequently reported in many countries worldwide [41,56,57].

In Saudi Arabia, our previous studies identified the subgenotype NA2 (genotype GA2) as the dominant strain in the winter seasons of 2007/08 and 2008/09 [29]. However, we reported a shift in the circulating subgenotype from NA2 to ON1 in the winter seasons of 2014/15 and 2015/16 [20]. The same genotype, ON1, was also detected in the winter seasons of 2019/20 and 2021/22 [30]. In the present study, we identified the ON1 subgenotype in the winter season of 2022/23. The rapid shift to ON1 and its stability over 13 years without the emergence of new genotypes/subgenotypes may be explained by the acquisition of novel antigenic features, particularly the duplicated region in the 2nd HVR of the G protein. The insertion of a duplicate region (60 nt/20 aa) was also reported in HRSV-B identified in Saudi Arabia in our previous studies [29,30]. This may help to resolve the HRSV to generate new genotypes and variants to cope with the host immune response. Further classification of ON1 into 4 lineages has been reported in several studies [16,58] and a fifth lineage has also been proposed [48]. In the current study, Saudi strains of the 5 winter seasons, 2014/15, 2015/16, 2019/20, 2021/22, and 2022/23, are scattered into 4 lineages (ON1-A to ON-D).

To understand the evolution and stability of the ON1 subgenotype, extensive analysis of the 2nd HVR, including the duplicated region (72 nt/24 aa), was performed. The ON1 strain in the present study presented 130 point mutations and 66 amino acid substitutions, whereas the ON1 strain in the winter seasons of 2014/15 and 2015/16 presented 112 mutations and 36 amino acid substitutions [20]. Among the 66 amino acid substitutions, 5 aa were changed to threonine, and 13 aa were substituted with serine, which consequently affected the glycosylation profile of the G protein. In the duplicated region alone, 18 amino acid substitutions were reported, and 6 amino acids (285Q, 288T, 292T, 295E, 302Q, and 306T) seemed to be unchanged across 13 years. In addition, Saudi ON1 strains have 3 specific amino acid residues at E232G, T253K, and 314 L, which distinguish them from other GA2 strains. Three specific sites were also observed in strains from China and Italy [59,60].

For the Riyadh strains from the winter season (2022/23), the duplicated region included 10 additional new O-linked glycosylation sites (288T, 291S, 292T, 293T, 294S, 299S, 301S, 305T, 306T, and 307S). The addition of such new glycosylation sites increased the number of potential O-glycosylation sites (67–82) in ON1 strains compared with other subgenotypes (56–77). The duplicated region does not affect the number of N-linked glycosylation sites; however, it results in a shift of N-glycosylation site 294 to position 318. Few N-linked glycosylation events have been reported in Riyadh strains (season 2022/23), which is slightly lower than those reported in the winter seasons of 2007/08, 2008/09, 2014/15, and 2015/16. Such variation in the N- and O-glycosylation sites combined with the change in protein length (imposed by the duplicated region) may increase the ability of the ON1 strains to escape preexisting immunity and cause recurrent infections [57,61,62,63]. Viral protein glycosylation reportedly has several functions, such as protein transportation, stability, the ability to mediate virus binding to cellular receptors, and the ability to hinder antibody binding [64]. Therefore, the increased number of O-glycosylation sites in the G protein of ON1 strains may explain the high incidence rate and increased risk of hospitalization [56,65,66].

The considerable mutation rate observed in the fusion (F) protein of HRSV-B strains, particularly within major antigenic sites, raises significant concerns about the effectiveness of vaccines based on the prototype HRSV A2 strain. The F protein is a primary target for neutralizing antibodies and, therefore, a primary candidate for vaccine development [67]. Its prefusion conformation is especially important, as it presents the most potent neutralizing antibody targets [68]. While the F protein is generally considered more conserved than the G protein, recent studies highlight the convergent evolution of mutations in the HRSV-B F protein, with key substitutions such as K191R, I206M, and Q209R becoming predominant in circulating strains since 2020 [69]. These mutations are located in or flanking major antigenic sites, including Ø, I, and V, suggesting a potential mechanism for immune evasion [70].

A comparative analysis of Riyadh isolates against the reference strain A2, which is commonly used for vaccine design, was conducted to identify mutations within the five major antigenic sites. At the nucleotide level, HRSV-A strains had 112 mutations, while HRSV-B strains showed a much higher number with 309 mutations. This significant difference in mutational load between the subgroups is consistent with previous findings of greater genetic variability in HRSV-B strains [67]. In the HRSV-A isolates, only 24 amino acid changes were found, with only three located in antigenic sites: two in Site Ø (K65R and R213S) and one in Site II (N276S). Notably, antigenic sites III, IV, and V were completely conserved in HRSV-A strains, indicating a high degree of stability, which is a favorable characteristic for vaccine targets.

These structural changes could alter the protein conformation enough to reduce the binding affinity of vaccine-induced antibodies, thereby diminishing the vaccine’s protective efficacy. HRSV-B isolates from Riyadh have 63 amino acid changes, with eight of these located within antigenic sites. Six of these mutations were found in antigenic site II, the palivizumab-binding epitope (D200N, K201N, K209Q, K209R, S211N, and N276S), with other changes including N67T (Site Ø) and I206M (Site II). This accumulation of mutations in HRSV-B antigenic sites, particularly Site II, highlights a significant difference in the evolutionary dynamics between the two subgroups and suggests a potential mechanism for immune evasion. The observation of multiple changes within the palivizumab-binding site (Site II) is of particular concern, as it suggests a potential for reduced efficacy of this prophylactic monoclonal antibody, a finding that warrants further investigation into contemporary circulating strains [70].

Since the current vaccine strategies depend mainly on the F protein from the A2 strain, the substantial genetic divergence of HRSV-B strains is a major challenge. While some studies have shown that vaccines based on the HRSV-A F protein can induce cross-protective immunity against HRSV-B, the level of protection may not be complete or equivalent [71]. For example, HRSV A-based monovalent Ad26/preF protein vaccine induced cross-neutralizing antibodies against both subtypes; however, the overall protective efficacy against HRSV-B was not always equivalent to that against HRSV-A [72]. This difference is likely due to the significant sequence divergence between the two subtypes [67]. Therefore, the accumulation of mutations in the F protein of circulating HRSV-B strains could indeed compromise the effectiveness of a monovalent vaccine based on the A2 strain, potentially leading to breakthrough infections and reduced overall vaccine impact in populations where HRSV-B is prevalent. This highlights the potential need for bivalent vaccines that include antigens from both HRSV-A and HRSV-B to ensure broad and robust protection [23].

## 5. Conclusions

This study provides comprehensive molecular surveillance of HRSV-A circulation in Riyadh, Saudi Arabia, confirming the sustained dominance of the ON1 genotype over multiple seasons, driven by constrained evolution in the duplicated region of the *G* gene and alterations in glycosylation profiles that likely enhance immune evasion. Critically, the F protein of contemporary HRSV-A isolates demonstrated remarkable antigenic stability, with only three amino acid substitutions mapped to antigenic sites Ø (K65R, R213S) and II (N276S), while sites III, IV, and V remained fully conserved. This high degree of conservation relative to prototype strains used in vaccine design (e.g., A2) supports the anticipated efficacy of recently approved prefusion F-based vaccines (Arexvy, Abrysvo) and the long-acting monoclonal antibody nirsevimab against HRSV-A in this population. In contrast, HRSV-B isolates exhibited substantially greater F protein variability, including multiple substitutions within the palivizumab-binding site II, raising concerns about reduced efficacy of this prophylactic agent and potentially incomplete cross-protection from HRSV-A-based vaccines. These findings underscore the importance of continued genomic monitoring to detect emerging variants that could compromise prophylactic interventions and highlight the potential value of bivalent vaccine strategies for broader protection. Overall, the preserved antigenic profile of circulating HRSV-A strains is encouraging for the implementation of current F-targeted preventives in Saudi Arabia.

## Figures and Tables

**Table 1:** Demographic characteristics and prevalence of HRSV-A in the study group.

		Number of Samples	Total Positive HRSV Samples	HRSV-A Positive Samples (%)	HRSV-B Positive Samples (%)
**Year**	2022/23	200	30 (15)	14 (7.00)	16 (8.00)
**Age in years**	<2 month	40	10 (25)	3 (7.50)	7 (17.50)
	2–5 months	89	10 (11.24)	6 (6.74) *	4 (4.49)
	6–11 months	45	6 (13.33)	3 (6.66)	3 (6.66)
	≥12 months	26	4 (15.38)	2 (7.69)	2 (7.69)
**Gender**	Male	117	18 (15.38)	9 (7.69) *	9 (7.69)
	Female	83	12 (14.46)	5 (6.00)	7 (8.43)

The data are displayed as numbers or percentages (%). Statistically significant values are labeled with * = (*p* < 0.05).

## Data Availability

The *G* and *F* gene nucleotide sequences for HRSV-A can be found in GenBank under accession numbers [G: PP454562–PP454570] and [F: PP454571–PP454579], respectively].

## Supplementary Materials

The following supporting information can be downloaded at: https://www.mdpi.com/article/10.3390/vaccines13121245/s1, Figure S1: Deduced amino acid alignments of the duplicate region. The figure shows the mutations observed in the duplicated region (42 aa) located in the 2nd HVR of the G protein of HRSV-A; Figure S2: Deduced amino acid alignments of the F protein. The 4 conserved amino acid substitutions in all strains are highlighted in red. The 5 conserved N-glycosylation sites are highlighted in blue. The red and blue triangles at the left of the figure refer to the consensus and Riyadh strains, respectively.
