# Supplementary material for: Molecular Surveillance Reveals F-Gene Mutations and Constrained G-Gene Evolution in Human Respiratory Syncytial Virus: Implications for Vaccine Efficacy in Saudi Arabia
Source: Vaccines (Basel). 2025 Dec 15;13(12):1245. doi: 10.3390/vaccines13121245 (PMC12737416; doi:10.3390/vaccines13121245)
Supplement: Supplementary file 1 [file vaccines-13-01245-s001.zip › vaccines-4027770-supplementary.docx]

**
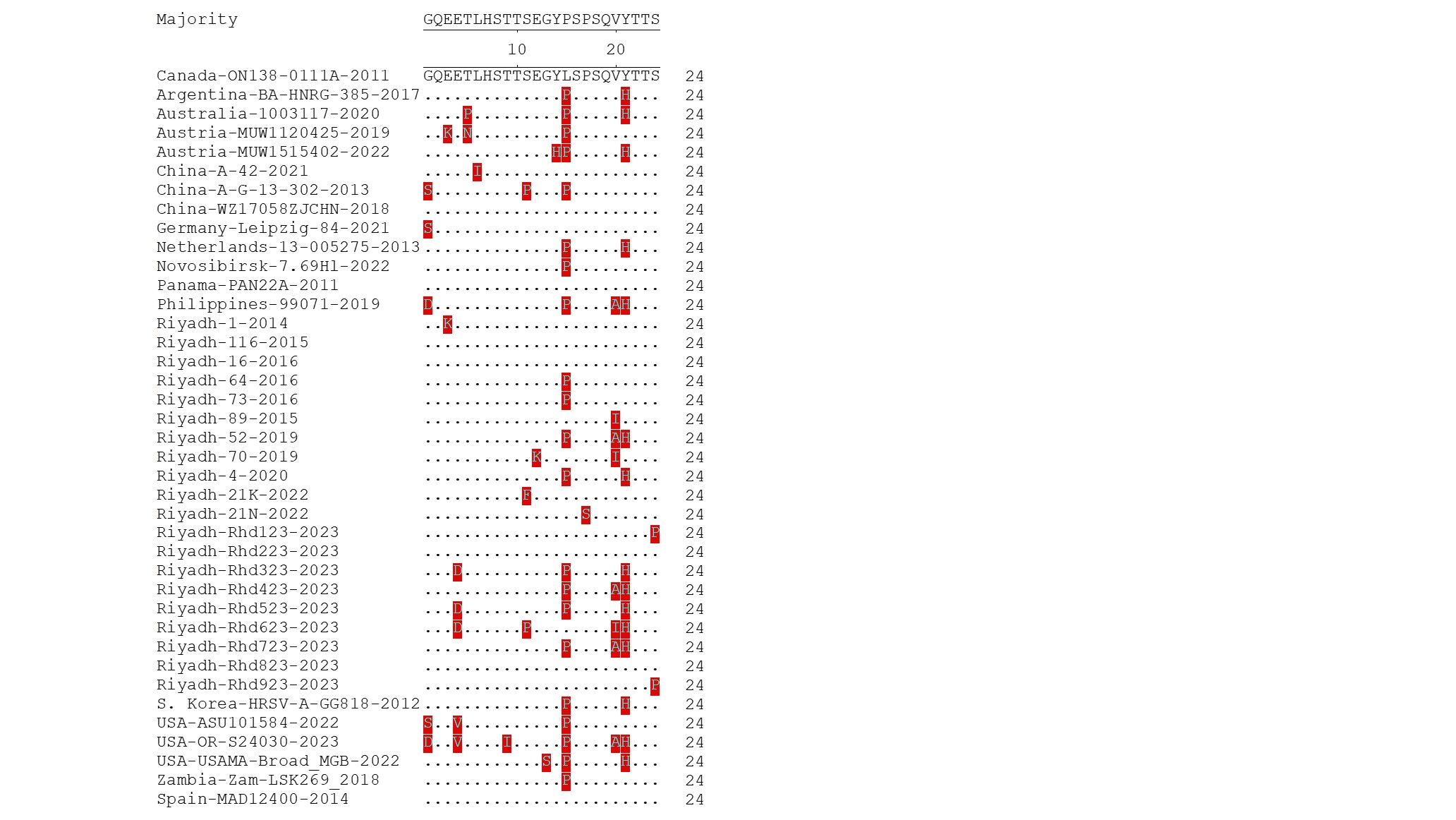
**

**Figure S1. Deduced amino acid alignments of the duplicate region.** The figure shows the mutations observed in the duplicated region (42 aa) located in the 2^nd^ HVR of the G protein of HRSV-A.


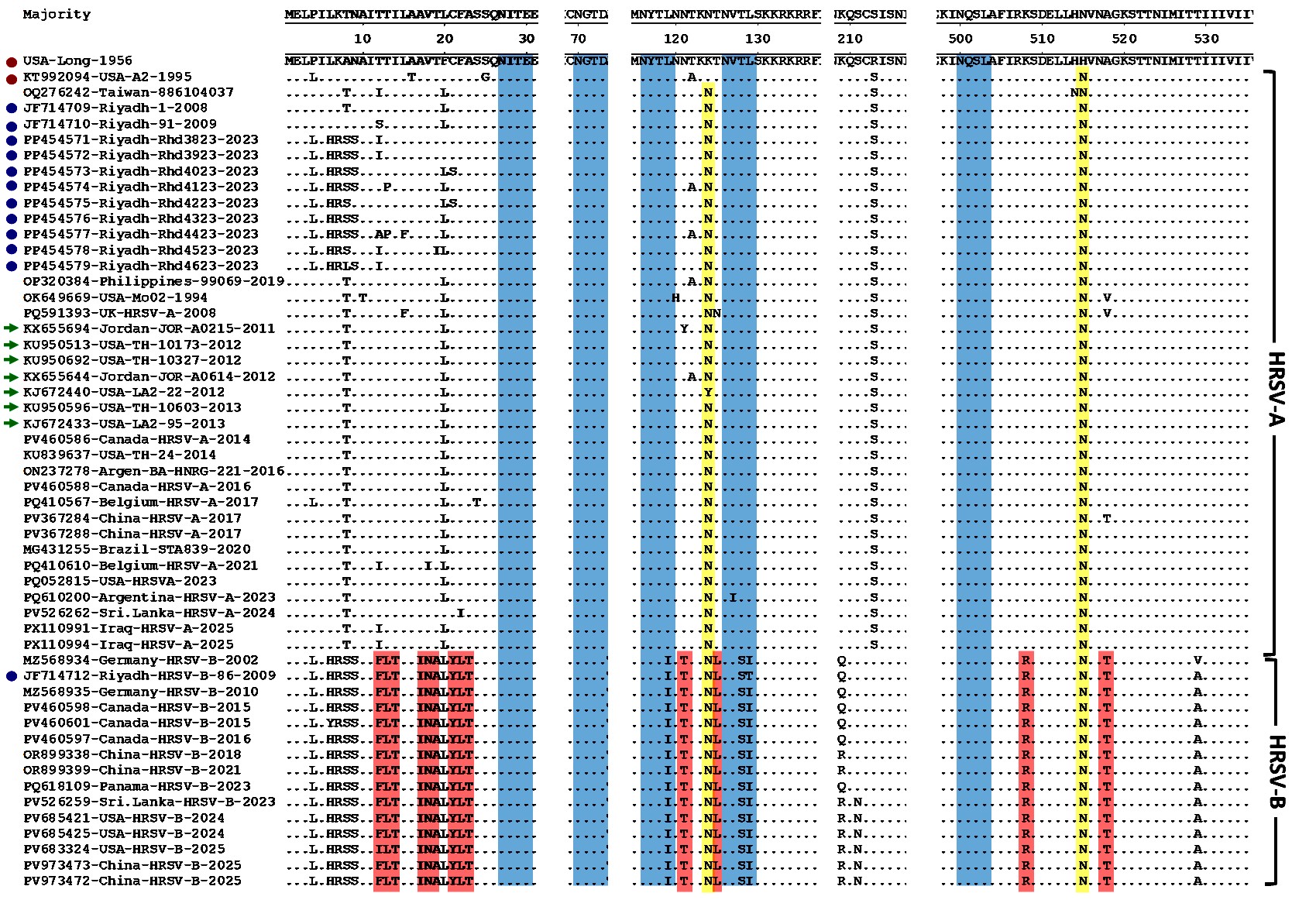
**Figure S2.** **Deduced amino acid alignments of the F protein**. The 4 conserved amino acid substitutions in all strains are highlighted in red. The 5 conserved N-glycosylation sites are highlighted in blue. The red and blue triangles at the left of the figure refer to the consensus and Riyadh strains, respectively.
